# Managers’ Perceptions of Barriers to Using IT for Organizational Development: Case of a Hospital Chain in Riyadh, Saudi Arabia

**DOI:** 10.1007/s44229-022-00023-w

**Published:** 2022-12-09

**Authors:** Maha Alkharji, Heba Alqurashi

**Affiliations:** 1 College of Health Sciences, Saudi Electronic University, Riyadh, Saudi Arabia; 2 Public Health Department, College of Health Sciences, Saudi Electronic University, Riyadh, Saudi Arabia

**Keywords:** Information technology, Health information system (HIS), Health information technology, Barriers, Effect, Organizational development

## Abstract

**Background:**

Information technology is widely used by managers in healthcare settings in Saudi Arabia and has positive effects on healthcare systems. This study focuses on mangers’ perspectives on barriers, as healthcare system users and leaders.

**Method:**

This work was conducted as a qualitative exploratory case study based on semi-structured audio-recorded interviews conducted at a hospital chain in Riyadh, Saudi Arabia, using open-ended questions to provide in-depth information.

**Results:**

The use of IT in healthcare settings has had positive effects, but many barriers can cause delays or problems in using IT, including hacking, human resistance to new technology and the need for experts in the field of IT in healthcare. Training and support of employees are leading solutions to the many problems encountered in implementing and using IT in healthcare.

**Conclusion:**

The study identified the problems in using IT for the development of healthcare services and the positive effects on the services provided to patients, and indicated a need for further development and support.

## Introduction

Information technology (IT)—defined as the process of receiving, sorting, processing, transferring and presenting information—substantially affects the rapid provision of systemic healthcare services [1]. Incorporating IT into any sector causes changes; for the healthcare sector to accept these changes, the benefits and challenges must be addressed. IT provides easy access to information and databases, to improve the quality of healthcare services, enhance information integration and facilitate decision-making for managers, while minimizing the risk of errors [2].

Research has addressed the positive effects of IT improvements in healthcare services. The healthcare sector is a dynamic field that is liable to undergo continual sudden shifts in learning and advancements. Anwar et al. (2011) have discussed how IT aids in communicating health-associated information effectively and efficiently among stakeholders through evidence-based decision-making processes in all fields, including medical and administrative aspects [3]. Moreover, Haghiri et al. have investigated how large volumes of healthcare information necessitate appropriate storage and management so that information is readily accessible to facilitate efficient and safe decision-making; enable high quality healthcare provision; and decrease possible errors, costs and paperwork [4].

Although, several studies have identified improvements in the quality of services in using IT systems, others have shown insignificant or negative effects on the quality of healthcare [7]. Owing to the importance of the use of IT in healthcare, further investigation must be conducted to examine the challenges and barriers, and provide insights and recommendations to maximize the benefits from IT use [8]. An example discussed by Khalifa (2013) has demonstrated how resistance from healthcare professionals, and a lack of training and expert support are barriers to the adoption of IT in healthcare [5]. Other barriers include financial aspects and staff shortages [3–6].

Hence, this study investigated the barriers to IT adoption in Saudi hospitals, as well as their effects on organizational development and performance improvement from the managers’ perspectives. It additionally explored solutions to enable IT to facilitate management of information and delivery of healthcare services.

## Literature Review

IT has markedly changed how healthcare operates, and appropriate application and implementation of health technology are critical for solving healthcare problems and providing improved healthcare services [6]. The use of IT in healthcare not only improves services but also decreases the potential for errors; many hospitals are continually adopting new technologies that link patients to hospitals and enable patients to access their medical information [4]. The ability to process large volumes of data and enhance the capabilities of databases in healthcare enables healthcare managers to develop better projections and make decisions in effectively allocating resources, channeling funds and acting on delivery plans at various administration, finance and clinical levels [2].

The Ministry of Health (MOH) in the Kingdom of Saudi Arabia has been implementing its National Transformation Plan for healthcare in alignment with the Vision 2030 initiatives, which focus on the digitization of healthcare and increasing connectivity, to ensure ease of access and standardization of health processes while providing high quality data and services [21].

In the literature, various barriers have been found to arise in the implementation of new technologies. The first barrier is the complexity of the implementation process, and the second is human factor barriers [1, 5, 6]. Each of these factors is discussed herein and is followed by suggestions from the literature on how to overcome them.

### Implementation Barriers

In the Saudi healthcare system, like many other governmentally funded healthcare systems, financial aspects are first addressed as the prime barrier, because the long approval processes of the legal authorities in the MOH can hinder the release of financial support or granting of requests [3].

Moreover, with the global focus on delivering quality services in healthcare worldwide, the second barrier addresses the importance of delivering effective and accurate output, and maintaining the confidentiality and integrity of the entire process of data recording and delivery; to accomplish these goals, a dedicated budget must be set and accompanied by the hiring and availability of appropriate staff [9].

The third barrier, a core element missing from the implementation process, is a shortage of staff with necessary IT knowledge and skills training [6]. Each hospital and healthcare institution competes to lead in the National Transformation Plan and promote technological change within the institution or facility [3, 6]. The last barrier addresses the provision of a system that is secure and protected from hacking or infiltration; this is a major priority in healthcare because of issues of privacy and confidentiality, and the importance of protecting health information [1, 6].

### Barriers Associated with Human Factors

The human element is a critical factor in any organizational change endeavor; however, it particularly important in the healthcare sector, in which it is an integral component and the most crucial barrier to the implementation of new technologies [2, 10].

The human factor can be explained through attitudes toward IT, and people’s ability to adapt to and learn to use a new system in daily work routines and in recording data systematically to enable the provision of better healthcare service. The main reason for resistance is the reluctance to change in transitioning from routine paperwork to the incorporation of a new technological approach [5, 9]. Hence, the first barrier for healthcare professionals’ is resistance due to the routine use of paperwork systems to which they have become accustomed, as compared with the required changes in work task routines and concerns regarding failure or lack of skills required for the adoption of new IT systems [1].

A second major impediment is associated with the attitudes of managers themselves, e.g., when they do not recognize the importance of this transformation and shifting toward incorporating technology into the organization, or when they are not engaged or are not mindful of the effort required to adapt to this shift [1]. Consequently, the third major barrier is communication within the organization or the lack of a common language, in addition to the lack of proper training of employees in communication with one another and with the IT experts, who are integral in supporting the transformation [1].

Finally, confidence and trust in the security of patient medical and health history and the confidentiality of data, which requires trusted and authorized individuals and systems to manage them, are top priorities in deciding on the workforce responsible for entering and managing medical and health data [9].

### How to Overcome the Barriers?

The literature has addressed several means of overcoming the aforementioned barriers of quality, trust, resistance and communication. The healthcare organization must keep pace with IT by aligning it to the quality of healthcare services; providing training and development programs for employees and medical staff on the use of IT systems; and understanding and addressing beliefs and attitudes through encouragement and communication regarding the importance of IT systems. These efforts can increase engagement and improve development strategies with the involvement of all stakeholders to overcome any problems [5, 11].

In addition, using an accurate IT system and a trusted workforce to address these data, as well as providing a qualified, easy, fast and workable healthcare IT system and support team, encourages the workforce to use the system [9]. For example, focusing on the user interface—such as its appearance, including colors, fonts and other features, and its functionality, including the ease and speed of data entry and extraction—can substantially encourage uptake or cause workforce resistance in engaging with the system, thus influencing the success of the system in supporting the delivery of healthcare services to patients, particularly those who require rapid treatment [9].

One way to increase communication is to involve all stakeholders in implementation, engage them with the concept, and address any issues or suggestions that they may have [9]. Moreover, to overcome the current barriers relating to finance and a shortage of staff and managerial support in Saudi Arabia, an important factor is the support of the MOH in facilitating the digitization of healthcare services in alignment with Vision 2030 [1]. To accommodate this change, the Saudi healthcare system is in being transformed to a fully automated system wherein finances, expert training, and development of qualified healthcare workers and administrative staff in use of this technology are critical [5].

## Methods

The current study used a cross sectional, qualitative, exploratory design, wherein the data were collected through case studies via semi structured interviews. The sample target was collected through nonrandomized sampling to achieve the research aims. The target was executive managers of hospitals with fully functional IT systems, accredited by the Central Board for Accreditation of Healthcare Institutions and the Joint Commission International.

The data were collected from four hospital executive managers in Riyadh, Saudi Arabia. Each interview was composed of ten questions, and the timing ranged from 15 to 30 min. The interview questions were initially pilot tested with three hospital executives and validated.

To enable data collection, ethical approval from the hospital institutional review board was received after approval from Saudi Electronic University on 05/05/2021 (SEUREC-CHS21122). Consent forms were signed by the interviewees to ensure confidentiality before the interviews commenced.

## Results

**Fig. 1:**
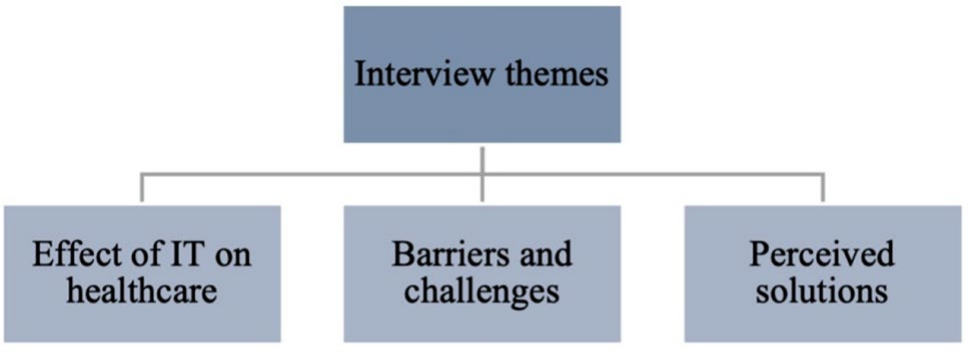
Interview themes

An exploration of the different perceptions of the interviewees was analyzed through thematic analysis, wherein each theme was displayed with its subcategory in accordance with descriptions during the interview, and was followed by an exploration of how each theme was derived and associated with the main theme. The subcategories of the interviews indicated three main themes: effects of IT, barriers and solutions (Fig. 1).

### First Theme: Effects of IT on Healthcare

**Fig. 2:**
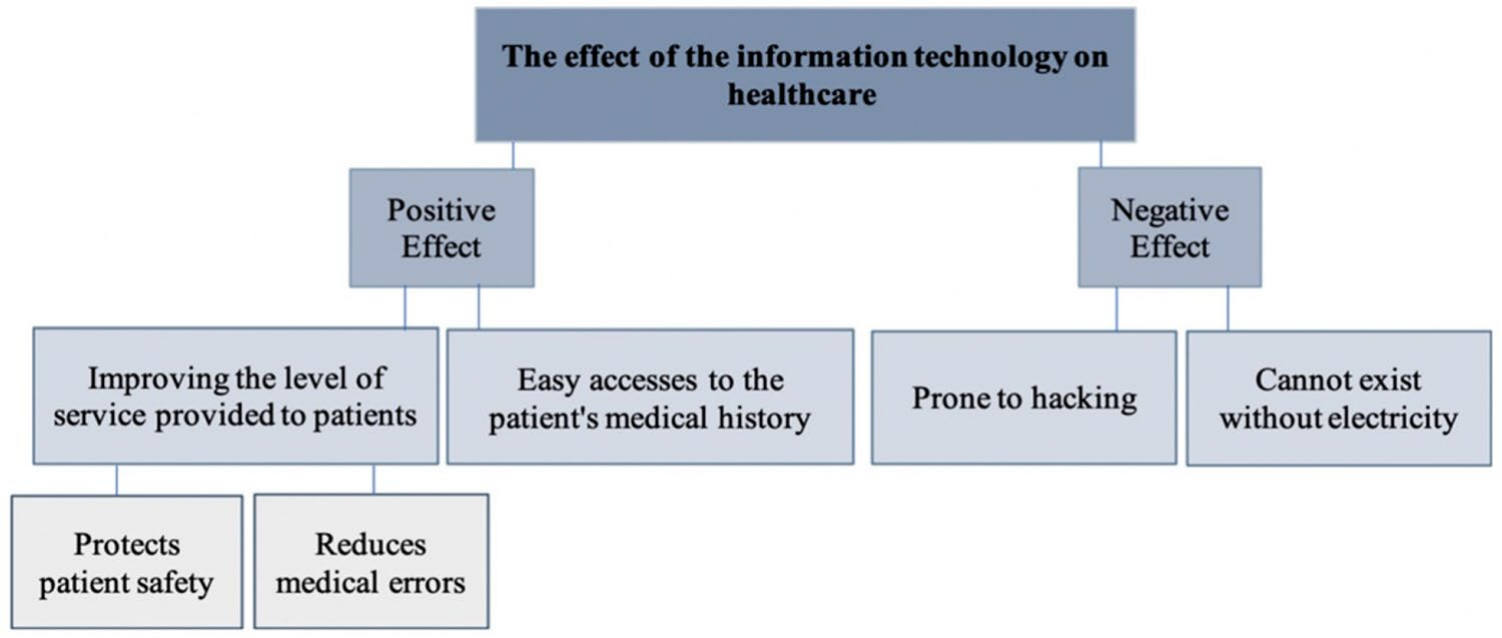
The effect of IT on healthcare settings

This theme was described by the respondents through two main subcategories: positive and negative effects (Fig. 2).

The first subcategory discussed positive effects through improving the level of service provided to patients and easy access to patient medical records (Table 1). The effects of IT on the level of service provided to patients was described by all respondents and is considered crucial for any healthcare institution.

**Table 1:** Positive effects of IT on healthcare through improving the level of service

Quote	Interviewee number
“The change from paperwork to the IT system has been beneficial in the flow of work … and has shown positive results in the work.”	Interviewee 2
“Using the IT system in entering the patient medical information is safer and reduces the errors resulting from writing and misreading.”	Interviewee 4
“In this time where we all face the COVID-19 pandemic, IT has limited the infection rate resulting from handing papers from one person to another.”	Interviewee 1

According to the interviewees, the positive effects were substantial, because of the importance of protecting patient privacy; ensuring the confidentiality of patient information; reducing medical errors through ensuring systematic data entry associated with correct coding mechanisms; and clinical decision support systems.

Additionally, the respondents recognized that, in the current context of the COVID-19 pandemic, IT has limited the incidence of infections arising from the handling of paper documents by different people.

Furthermore, the second positive effect of IT was ease of access, which not only decreased medical errors but also aided in the systemization of the service delivery, thus allowing for less time spent away from patients while checking their history, and more time dedicated to patients and discussion of their current condition (Table 2).

**Table 2:** Positive effects of IT on healthcare through ease of access to patients’ files

Quote	Interviewee number
“After applying the IT system, as soon as the doctor enters the mobile number of the patient in his clinic, he can see the patient's information, which helps him in performing his work and reduces the percentage of medical errors.”	Interviewee 1
“Application of the IT system protects patient privacy and reduces the medical errors produced from using oral and paper contacts. It made access to the patient information easy for all the branches that facilitate the reading of the information, decision-making and the mechanism of work. Before, reading handwritten files could cause medical errors and sometimes double entry of patient information. IT contributed to reducing these errors, and ensured the safety and confidentiality of the patient data. It also saved the space needed for archiving the paper files.”	Interviewee 3

However, respondents also mentioned negative effects of IT, and discussed the possibility of hacking while the system is active or loss of data when electricity is not available. According to the respondents, hospitals, like other systems, are prone to hacking; therefore continual updates of security measures and ensuring continual changes to access are critical (Table 3). Therefore, most hospitals incur costs for ensuring the acquisition of a highly protected system with a strong firewall that is consistently updated.

**Table 3:** Negative effects of IT on healthcare through hacking

Quote	Interviewee number
“We changed the whole environment of work. This has cost us a lot financially, and providing an experienced IT team was a challenge. But the final result was worth it.”	Interviewee 1
“Our system is highly secured from hacking, as we understand the importance and confidentiality of the patient data. We have a highly skilled and active IT team who applied a firewall and antivirus system that has protected our data from hacking, especially in the last two years, when hacking activities have increased. So our IT team always seeks to update this system all the time.”	Interviewee 1

Interviewees 3 and 4 indicated that they faced issues of losing access to information because of a power outage, and the hospital had to switch back to using a paper system until the problem was resolved. Checks were made to ensure that the system was ready and safe to be used again (Table 4).

**Table 4:** Negative effects of IT on healthcare due to electricity failures

Quote	Interviewee number
“IT disadvantages are mainly that it is prone to hacking, and it is closed once the electricity is gone. And when this happens, we return to the paperwork till the problem is solved, and make sure the system is ready and safe to be used again. We have a highly protected system, but, like any other system, it is prone to hacking which is constantly changing. Our firewall is always updated on the financial and technical level.”	Interviewee 3
“As we are a healthcare facility providing services to patients who in the first place need medical attention and care, we will feel disturbed and anxious when we are shifting from the IT system to paperwork during system problems. And even for the employees, they find it difficult to shift to paperwork even if they have a background and previous work experience with paperwork, but this is a load on them”	Interviewee 4

Hence, the IT system's application positively affected the medical and administrative staff in providing services and improving patients’ experiences through protecting their privacy and reducing medical errors. However constant fears regarding loss of data, due to external factors or power outages, persist.

### Second Theme: Barriers and Challenges of IT in Healthcare Settings

**Fig. 3:**
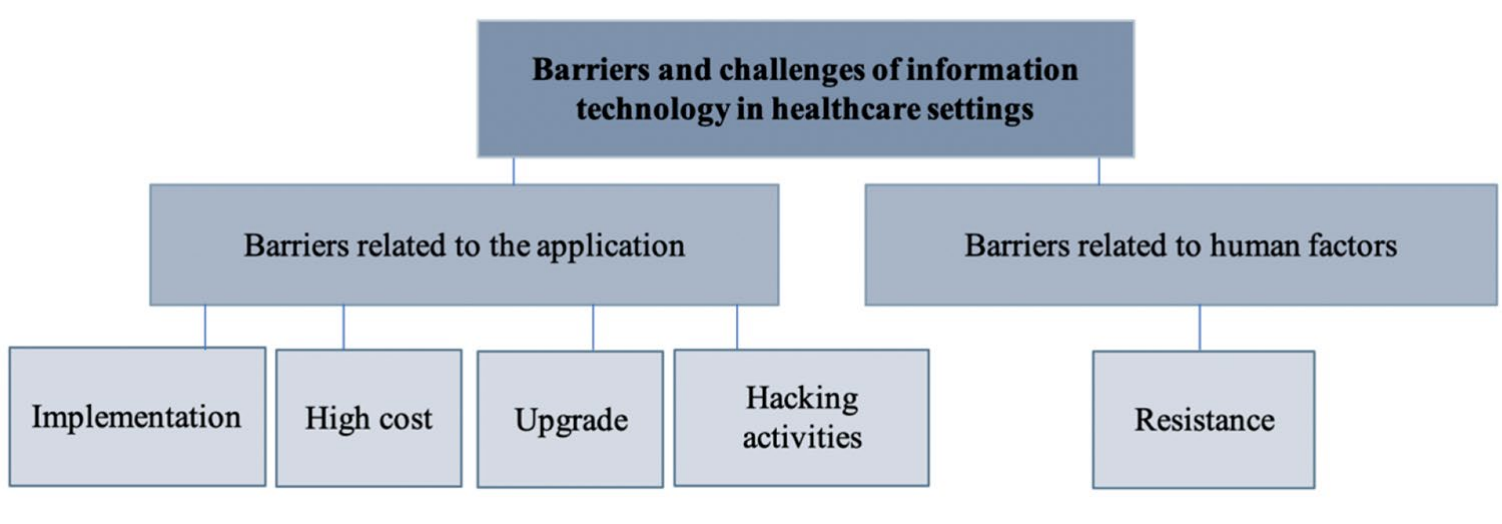
Barriers and challenges of IT in healthcare settings

The second theme (Fig. 3) addresses the barriers and challenges described by the interviewees, divided into two main subcategories relating to the IT application or to human factors.

According to most interviewees, the most challenging stage in applying the IT system was the automation of all paperwork processes, which required substantial time, effort and cost. Thus the initial implementation of the system is critical and must meet all requirements, including ensuring continual updates, daily security checks, and provision of a user-friendly system that allows information to be obtained safely and securely (Table 5).

**Table 5:** Barriers to IT implementation in healthcare settings due to IT implementation

Quote	Interviewee number
“The most difficult stage in application of the IT system was the entering of the old data into the system.”	Interviewee 2
“The most difficult part we faced when we changed from paperwork to the IT system was entering the old data automatically or manually. That cost us time, effort and money. The difficulty of this process depends on the management of the healthcare facility and how it promotes the financial and moral support till the complete transition to IT system.”	Interviewee 3
“As the organization grows, the support for the automation will be harder. So the first implementation of the system is very important and has to meet all the needs that make updating easy and smooth.”	Interviewee 4

In contrast, barriers associated with human factors were also indicated. Although human resistance from younger employees was not encountered, it was present among older healthcare workers. The most resistance was observed in the implementation phase, mainly among medical physicians (Table 6).

**Table 6:** Barriers to IT implementation in healthcare settings due to human factors

Quote	Interviewee number
“This is not likely to happen in the era of technology, where all have a background in using technology and know how much it made our life fast and easy; the only resistance maybe comes from old, aged persons who are still attached to the old ways, but this is very rare.”	Interviewee 1
“The resistance rises from the doctors, especially those who are older and are not used to this technology. The resistance was in the time the doctor consumed at the beginning of using the system.”	Interviewee 4
“Resistance is a part of human nature. But soon this resistance disappears after the employees know how easily they can use the system, and how much it will save their time and effort.”	Interviewee 2
“There was resistance from the workforce, as resistance to change is human nature.”	Interviewee 3

Barriers are crucial in the introduction of new approaches, and human resistance to change is a natural process that must be addressed and observed. The use of new technologies also requires that various resources be implemented and maintained throughout the course of use.

### Third Theme: Solutions to Addressing Obstacles and Challenges of IT in Healthcare Settings

**Fig. 4:**
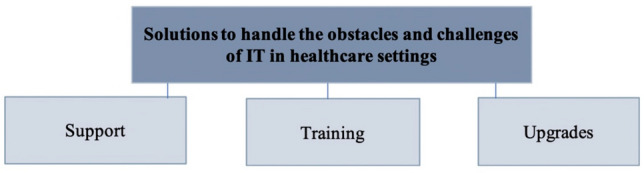
Solution to handle the challenges of IT in healthcare settings

All the interviewees suggested solutions to aid in the automation of healthcare settings (Fig. 4), through three main subcategories: support, upgrades and training.

The first subcategory, support, was classified by the respondents into support from the organization and support from the MOH (Table 7). Organization support was discussed differently among the four interviewees: interviewees 1, 2 and 3 stated that providing financial and moral support continually motivates people to use the new IT system, whereas interviewee 4 stated that, at his organization, support is considered a proactive process before, during and after the implementation of the IT system.

**Table 7:** Solutions for IT implementation through support

Quote	Interviewee number
“The workforce has been supported financially and morally to use the system, and followed up step by step until the IT system’s complete transition.”	Interviewee 2
“Support for the transition occurs when the staff see how the IT system improves their work and how it changes it for the better; it occurs proactively, where the manager knows before the application of a new system that he must deliver the change smoothly and show support for his employees.”	Interviewee 4
“The Ministry of Health has shown full support in applying the IT system; even the periodic follow-up by the MOH on hospital files has become easy and fast. Instead of reviewing paper files, all they need is to enter the database and review all the digitally documented data.”	Interviewee 1

All interviewees agreed that support from the MOH in facilitating the implementation of Health Information Technology though IT in healthcare has increased the standards and quality of service provided for patients and has facilitated periodic follow-up by the MOH.

Consequently, all respondents stated that training is of major importance in allowing for ease of use, including training for new employees and system upgrade training (Table 8).

**Table 8:** Solutions for IT implementation through training and upgrades

Quote	Interviewee number
“Training, whether on any new updates on the system or when a new member joins the hospital, is essential to overcome the barriers.”	Interviewee 2
“Continuous improvement and updating are critical to set the technology on the right path, and constant updates of the system eliminate vulnerabilities that can be exploited by hacking techniques.”	Interviewee 4

Accordingly, the solution subcategories addressed the previous problems that the interviewees faced; all respondents agreed that, for an IT system to be implemented correctly and to be accepted by employees, choosing a qualified system, ensuring its continual updating, and providing routine training and support to employees are essential.

## Discussion

The results showed that all respondents agreed that the effects of IT use in healthcare are positive, by improving the quality of services provided to patients by reducing medical errors and increasing the security and confidentiality of patient information, as supported by many researchers. The main themes of the interview results supported prior literature findings and the objectives of the current research.

Although IT systems are implemented to improve the quality of healthcare services, the safety and confidentiality of these systems was highly important to the interviewed hospital managers, as described by Interviewee 1:

*"Our system is highly secured from hacking, as we understand the importance and confidentiality of patient data. We have a highly skilled and active IT team who applied a firewall and antivirus system that has protected our data from hacking, especially in the last two years, when hacking activities have increased. So our IT team always seeks to update this system all the time."*

Hacking of hospital systems was indicated as an issue by all managers, and patient confidentiality and information protection were of high priority; consequently, the managers agreed that a strong firewall and security system would help circumvent this issue [2, 6, 12–14].

Moreover, the results showed two effects of the use of IT in healthcare settings, wherein the outcomes of the system on healthcare services are directly affected by the quality of the system in either a positive or negative manner. One of the negative effects is the increase in the implementation costs. According to Mohamadali and Ab Aziz (2017), these costs have long-term benefits, as the system and technology are relatively new; various studies have also indicated that IT use in health settings is new and needs support, but have not referred to costs [4, 6, 9].

According to various studies, IT systems in healthcare have greatly increased patient safety, and are an excellent tool for healthcare providers to track patient health, provide high-quality care and integrate clinical data analysis to prevent medical errors [13–17], as supported by the results of this study.

The security of data transfer and patient information should be ensured, and measures for this purpose should be imposed through the careful selection of trustworthy employees in each department who access patient records throughout the transformation process and subsequently [9].

Furthermore, the focus on training as a solution has also been confirmed in the literature: qualifications and training have been reported to be crucial in overcoming barriers to the application of IT in health facilities [3, 9, 13, 16, 18], and the consistency between human supplies and process aspects enables IT to perform efficiently [19].

The barrier of physicians' resistance was stated by the interviewees, who recommended assisting employees in understanding the importance of healthcare information technology, to improve the services provided. Other researchers have discussed how changing the typical recording from paperwork to software databases might be resisted by physicians, particularly those who are older, and have indicated that the changes in adopting new technology must be presented smoothly before the implementation of new systems, and that individual and usage support must be provided [5, 14, 18, 20].

All respondents stated that the MOH had shown full support in applying IT systems, thus increasing the standards and quality of services provided to patients. In contrast, Sadoughi et al. (2017) have reported that complications in gaining approval from the legal authorities of the MOH to promote health IT are increasing the gap between planning and introducing IT to healthcare systems, and hindering successful implementation to achieve the desired results [6].

Finally, Saudi’s eHealth strategy has essential elements that are health sector-wide and will link the MOH, other government services and private sectors for all health systems. The Saudi Health Information Exchange will also enable sharing of all health data among hospitals in Saudi Arabia, and will be an essential mechanism for integrating digital capabilities across all parts of the health system [22, 23]. Hence, the MOH encourages private facilities to adopt electronic transformation in proceeding along this path [6].

### Limitations

The study was conducted during the height of the COVID-19 pandemic, thus significantly decreasing the amount of available time that healthcare managers had to participate in the research. Additionally obtaining permission from the hospitals to conduct the study was challenging, owing to the safety restrictions.

## Conclusion

The study has merit because the use of IT in healthcare settings in Saudi Arabia is considerably new, and understanding the barriers to IT use will help improve the utilization of IT to provide better healthcare services to patients. We found that the need for experts in the field of IT in healthcare is essential for the correct implementation of IT in hospitals.

This research highlighted the value of IT by indicating the substantial benefits of the system in reducing wasted time and human errors, and substantially influencing the services provided to patients. Furthermore, IT aided in the scaling of services to levels that cannot be achieved with traditional manual systems in hospitals, by linking all hospital systems—including imaging, financing, medical, clinical and managerial aspects—thus enabling faster, more effective evaluation of patient cases, and leading to provision of better healthcare services.

Furthermore, the resistance to new technologies by employees, and fears regarding patient data security and privacy are major issues facing IT use in healthcare in Saudi Arabia. For example, hacking from outside the healthcare organization, accessing the data without authorization, or selling data to other institutions or even other industries that may benefit from patient data are other barriers in need of attention.

The obstacles described herein apply not only in healthcare settings but also in sectors that use technology worldwide. Thus, the solutions proposed could be applied to all sectors. These recommendations include continual firewall updating, particularly for personal, financial and managerial data, to help overcome fears of hacking. Moreover, hospitals should use these tools and update them regularly to ensure the safety, security and privacy of patient data.

## Abbreviations

IT: Information technology
IS: Information systems
MOH: Ministry of Health
KSA: The Kingdom of Saudi Arabia
NTP: National transformation plan
CBAHI: Central Board for Accreditation of Healthcare Institutions
JCI: The Joint Commission International
IRB: Institutional Review Board
HIS: Health information systems
SeHE: Saudi Health Information Exchange

## Declarations

### Availability of Data and Materials

Data sharing is not applicable to this article, because no datasets were generated or analyzed during the current study.
